# Emotional competence and help-seeking intentions as predictors of educational success in vocational training students

**DOI:** 10.1177/14779714241265463

**Published:** 2024-07-24

**Authors:** William Gilbert, Dale M Stack, Erin T Barker, Annie Dubeau, Lisa A Serbin, Marie-Hélène Véronneau

**Affiliations:** Department of Health Sciences, 14846 Université du Québec à Rimouski, Rimouski, QC, Canada; Centre for Research in Human Development, 5618 Concordia University, Montreal, QC, Canada; Department of Psychology, 5618 Concordia University, Montreal, QC, Canada; Centre for Research in Human Development, 5618 Concordia University, Montreal, QC, Canada; Department of Psychology, 5618 Concordia University, Montreal, QC, Canada; Centre for Research in Human Development, 5618 Concordia University, Montreal, QC, Canada; Department of Education and Specialized Training, 14845 Université du Québec à Montréal, Montreal, QC, Canada; Department of Psychology, 5618 Concordia University, Montreal, QC, Canada; Centre for Research in Human Development, 5618 Concordia University, Montreal, QC, Canada; Department of Psychology, 14845 Université du Québec à Montréal, Montreal, QC, Canada; Centre for Research in Human Development, 5618 Concordia University, Montreal, QC, Canada

**Keywords:** adult learning, emotional intelligence, help seeking behavior, social emotional competence, vocational education

## Abstract

Given the high prevalence of psychological distress among vocational training (VT) students, this study aimed to assess the role of interpersonal emotional competence as a resilience factor promoting the educational success of this population. We postulated that emotional competence would promote educational success, both directly and indirectly by fostering students’ help-seeking intentions when facing a personal or school-related problem. To test these hypotheses, we used a sample of 219 VT students from the Canadian province of Quebec (68% women, M_age_ = 24.58; SD_age_ = 7.95) enrolled in various programs (e.g. institutional and home care assistance, welding and fitting, secretarial studies, and professional cooking). These students were assessed two times, during the first half of their training and again after their training. Results from structural equation modelling revealed that emotional competence was a positive predictor of help-seeking intentions and educational success. However, having the intention to seek help did not translate into higher levels of educational success. Overall, these results highlight the importance of supporting VT students in the development and strengthening of their emotional competence to promote their educational success. Future research is needed to further understand the help-seeking process among VT students and its implications for their academic outcomes.

## Introduction

Demographic changes in the western world are raising major concerns relative to labour shortages in sectors that are crucial for maintaining the well-being of the population and for fostering economic growth (Fuller, 2015; Lund & Karlsen, 2020). Indeed, health, construction, and manufacturing sectors are struggling to find qualified workers to handle medical equipment, provide healthcare, and maintain public infrastructures (OECD, 2022; Statistics Canada, 2019, 2022). Vocational training (VT) programs, also known as trade schools, could be an important part of the solution towards tackling this ongoing labour crisis as they typically provide well-structured and specific hands-on skills training taught in a short amount of time. Students can earn a VT diploma in as few as 6–18 months (depending on each specific program) – as long as training centres are able to ensure the perseverance and educational success of their students. Unfortunately, a significant proportion of VT students suffer from psychological adjustment problems that threaten their success (Carroz et al., 2015), and research examining the psychological and social protective factors that could be targeted for intervention with this population is lacking. To fill this gap, this study aims to assess how emotional competence and help-seeking intentions can contribute to fostering VT students’ educational success.

### The vocational training system

VT programs exist all over the world, but their structure and implementation can vary considerably from one country (and even region) to another.^1^ In most countries, these programs are divided into two categories according to the proportion of training provided at school and in the workplace: school-based programs and combined school/work programs (the latter category is also referred to as apprenticeship programs; OECD, 2019). In certain regions and countries, such as the Canadian province of Quebec where this study was conducted, VT programs are an integral part of the educational system (Ministry of Education and Higher Education [MEHE], 2019). These programs provide qualified workers in a variety of domains, some of which are highly specialized as a function of geographic areas across the province, especially in rural regions (e.g. fisheries, mining, and agriculture). They also provide secondary-school-level qualifications to youth who may struggle to succeed in traditional secondary school (i.e. secondary school in Canada begins in Grade 7 and ends in Grade 11) and who learn better with hands-on training (Thouin et al., 2023). Therefore, Quebec’s Ministry of Education has developed a VT system that is accessible to a wide range of prospective students. Youth can access VT programs after fulfilling the basic requirements of the third or fourth year of the general secondary school program (i.e. Grade 9 or 10). Individuals who do not possess such credentials but who have relevant work experience or foreign diplomas can also engage in a VT program by proceeding through the credential recognition process (MEHE, 2005).

### Educational success for vocational training students

Although the primary mandate of VT schools is to ensure that students obtain their course credits and diploma within a specific timeline, the Ministry of Education’s *Educational Success Policy* (2017) also requires that schools teach students a set of transferrable skills to be used in actual job settings and to become responsible citizens within their community. The global mission of Quebec’s school system is to provide instruction, qualification, and socialization to students. *Instruction* refers to stimulating students’ intellectual growth and cognitive development by helping them acquire and master meaningful knowledge. *Qualification* refers to guiding students in the acquisition of the practical skills needed to achieve their professional goals and find a job aligned with their field of study. Lastly, s*ocialization* is about developing students’ democratic values and community engagement. It refers to supporting the development of their sense of belonging to society and helping them learn how to live and work with others despite individual differences. Elementary, secondary, and post-secondary schools in all sectors are responsible for carrying out this broad mission.

To date, grades and graduation have been used as the primary indicators of students’ educational success in research efforts, mainly serving to inform on the instruction component described above (i.e. acquisition of knowledge, Dubeau et al., 2017). As a result, the qualification and socialization components have been largely neglected, thus resulting in an incomplete portrait of the many aspects that characterize VT students’ educational success. To address this shortcoming, Tsakpinoglou and Véronneau (2022) developed a questionnaire assessing students’ perceptions of how their VT program contributed to their instruction, qualification, and socialization. These authors proposed that considering these various dimensions, either individually or globally, should lead to a more comprehensive and inclusive assessment of VT students’ educational and professional development as well as its crucial determinants.

### Psychological adjustment of vocational training students

Achieving the educational goals described above can represent a major challenge for schools when students experience significant difficulties in terms of psychological adjustment. Numerous studies suggest that VT students present higher levels of adjustment difficulties than those in general secondary programs or in post-secondary education (e.g. Carroz et al., 2015; Drolet, 2017). In a study conducted among over 2600 VT students (Beaucher et al., 2021), 28.5% reported significant psychological distress according to Kessler et al.’s (2002) screening instrument. Results from the study also revealed that 13.8% of participants used alcohol and 21.2% used drugs at least three times per week. To verify whether VT students experienced more mental health issues than the general population, another study recruited both VT students and a sample of normative adults from a similar age range, including mostly workers but also students in programs other than VT (e.g. university programs), and a minority of participants with other occupational statuses (e.g. retired; Montminy et al., 2023). After controlling for participants’ gender and age, this study corroborated that VT students used more cannabis and tobacco than participants in the normative sample. Moreover, female VT students presented higher levels of anxiety and depressive symptoms than women in the normative sample. Considering that such psychological difficulties are related to academic failure (Sharp & Theiler, 2018), it is crucial to identify malleable resiliency factors that can foster VT students’ optimal functioning during and after their training. In this context, emotional competence and help-seeking skills are likely to play a major role.

### Emotional competence and help-seeking

Emotional competence, originally introduced as ‘emotional intelligence’ (Salovey & Mayer, 1990), is a set of skills that enable individuals to manage their emotions strategically to achieve significant personal and social goals (Denham et al., 2015; Saarni, 1999). Since the formal introduction of this concept by Salovey and Mayer (1990), several studies have been conducted to better understand the nature and implications of emotion-related individual differences. In this study, we adopt the trait conceptualization of emotional competence which postulates that individuals have a propensity to deal with their emotions and those of others in ways that are more or less adaptative (Mikolajczak, 2010). Based on this trait conceptualization, Brasseur et al. (2013) proposed that emotional competence encompasses five categories of skills. The first two categories are emotion identification and comprehension, which refer respectively to the ability to perceive and identify emotions, and the ability to understand and discern their causes and consequences. The next categories are emotion expression and utilization which, respectively, reflect the ability to adequately express emotions and to use them to improve reflection, decisions, and actions. The last category is emotion regulation, which refers to the ability to regulate emotions upwards or downwards depending on the context.

The importance of these emotional skills transcends many life domains, including education. For instance, among secondary school students, emotional competence has proven to be a key skill set to foster school success (Alzahrani et al., 2019) and also to improve school performance among adolescents with academic difficulties (Petrides et al., 2004). Among first-year university students, a study showed that those who obtained a high end-of-year GPA (equivalent to 80% or better) were more highly skilled in emotion identification and regulation than students who obtained a weak GPA (equivalent to 59% or less; Parker et al., 2004). Emotional competence has also been shown to predict young adults’ educational attainment, beyond well-known predictors such as socioeconomic status and school grades (Véronneau et al., 2014; Keefer et al., 2018). More broadly, the ability to receive and use constructive criticism, to express oneself in an appropriate tone, and to regulate oneself when experiencing intense emotions is considered crucial for maintaining adequate interpersonal interactions during one’s training, and these abilities are usually expected by employers who will hire students once their training is completed (Guerra et al., 2014).

To better understand the role played by emotional competence in the prediction of students’ academic outcomes, it is relevant to distinguish between the intrapersonal and interpersonal dimensions of emotional competence (Brasseur et al., 2013; Mikolajczak et al., 2009, 2014). The intrapersonal dimension, which refers to an introspective use of emotions, is valuable in helping students find their vocation, as individuals who attend to, understand, and use their emotions to make important decisions may improve their self-knowledge and facilitate their career choice. In contrast, the interpersonal dimension is crucial to developing and maintaining rich and positive social relationships that are essential for navigating an academic program and for integrating harmoniously into the labour market (Mikolajczak et al., 2014). More precisely, the interpersonal dimension of emotional competence is expected to be particularly important for educational success given that it was shown to promote help-seeking intentions which are major determinants of help-seeking behaviours (Ciarrochi et al., 2003; Keefer, 2015; Nagai, 2015).

Help-seeking intentions might be particularly crucial for VT students given their high propensity of experiencing psychological adjustment difficulties combined with the intensive structure of their VT program which requires many hours of coursework and internship training each week with limited holidays throughout the program duration. Accordingly, prior research raised the importance of focusing on students’ help-seeking tendencies by highlighting potential deficits in this area. For instance, Beaucher et al. (2021) reported that most VT students believe that they can count on their family (86.6%) and friends (67.7%) when they need social support. However, although these individuals may be well positioned to offer support for a range of personal issues, they may not be able to provide adequate support in the case of academic difficulties. Yet, only 28.4% of participants identified staff members from their VT centre as a potential source of social support (Beaucher et al., 2021). Other studies conducted in universities showed that an important proportion of students do not seek formal help for their academic difficulties or mental health problems (Hunt & Eisenberg, 2010; Shin & Jeon, 2011). These results highlight that some students are more reluctant than others to seek help, especially from professionals, which could seriously threaten their ability to successfully complete their training and impair their readiness to enter the labour market. As social support was shown to be positively related to professional identity, employability, and educational success (Chen et al., 2023; Tentama et al., 2019), more research is needed to find ways of increasing VT students’ willingness to ask for help when they need it.

### The present study

Despite the potential benefits of emotional competence for VT students’ educational success and professional development, little is currently known about the correlates and outcomes associated with specific dimensions of emotional competence among this population. Based on the results reported above, the interpersonal dimension of emotional competence could be particularly important for VT students by facilitating different stages of the help-seeking process in the event of personal or academic needs. This assumption, however, has not been tested in previous research.

**Figure 1.**
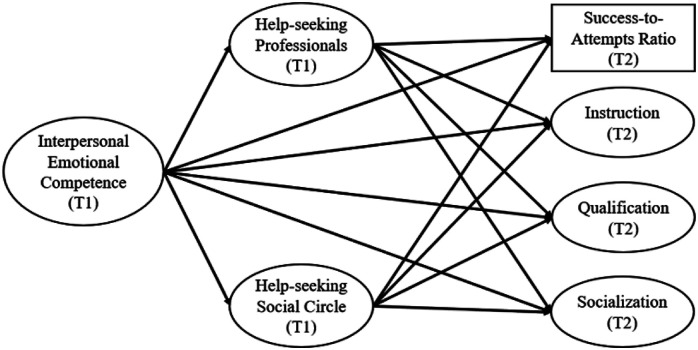
Illustration of the prediction model tested in this study. *Note*. Sex was added in this model as a control variable in the prediction of educational success, but it is not illustrated in the figure for simplicity. Ovals: factor scores; rectangle: manifest score; T1: Time 1; T2: Time 2.

Based on these shortcomings, the present study aims to assess the roles of emotional competence and help-seeking intentions in predicting VT students’ educational success. To do so, we rely on a multidimensional assessment of educational success encompassing students’ instruction, qualification, socialization, and objective academic performance (see Figure 1). Based on previous research (e.g. Alzahrani et al., 2019; Petrides et al., 2004), we postulated that students’ interpersonal emotional competence as measured during the first half of their VT program (Time 1; T1) would positively predict their levels of educational success at the end of their program (Time 2; T2) (Hypothesis 1). According to previous findings (e.g. Chen et al., 2023; Tentama et al., 2019), we also postulated that VT students’ intentions to seek help from members of their social circle or from professionals when facing a personal or a school-related problem, as measured at T1, would positively predict their levels of educational success at T2 (Hypothesis 2). Lastly, given the role played by emotional competence in facilitating the help-seeking process (e.g. Ciarrochi et al., 2003; Keefer, 2015), we postulated that help-seeking intentions would mediate the relationship between students’ interpersonal emotional competence and their educational success (Hypothesis 3). While testing these hypotheses, we controlled for the effects of sex on educational success given that previous studies have repeatedly shown that women outperform men in terms of academic achievement (Barry, 2019; OECD, 2015b) and because women and men in VT have different profiles (e.g. they attend different types of programs, and women in VT are usually older than their male counterparts; de Larquier & Remillon, 2022). This study will provide important insights regarding the malleable psychosocial factors that can promote the educational success of VT students, an important endeavour given the current labour shortages in major sectors of activity.

## Method

To test our hypotheses, we relied on a longitudinal design in which a sample of VT students was recruited in different VT schools to complete a questionnaire during the first half of their studies (Time 1) and again after graduation (Time 2). The data collected from these two time points were then used to perform structural equation modelling. This sophisticated statistical approach enabled us to analyze the relationships between interpersonal emotional competence at Time 1 and educational success at Time 2, while evaluating whether help-seeking intentions (Time 2) contributed to explaining these relationships.

### Procedure and participants

A sample of 219 students (149 women and 70 men) aged between 16 and 59 (*M* = 24.58; *SD* = 7.95) was recruited in 10 VT schools administered by four school boards in the Canadian province of Quebec. Of these 219 students, 75.3% declared that they were born in Canada and 74.4% declared French as their mother tongue. This sample is representative of the Quebec VT student population in terms of sex and age. Indeed, although this sample included many types of programs (e.g. welding and fitting, administration, accounting, and professional cooking), those in which emotional skills are particularly relevant (e.g. care sector) were intentionally overrepresented in this research project, and those programs overwhelmingly include women, hence the gender imbalance in our sample. Moreover, close to 50% of VT students in Quebec are aged between 20 and 29 years old, which is aligned with the mean age of our sample (MEHE, 2023).

Recruitment was carried out by research assistants who visited students during class hours. Data were collected at two time points, while students were in the first half of their training (Time 1, *N* = 219) and on average 5 months after the end of their training (Time 2, *N* = 134). At T1, students were asked to complete a paper questionnaire. At T2, we contacted participants by mail, phone, text, or email to invite them to complete an online questionnaire. This project received the approval of the Research Ethics Review Board from the last author’s institution. At both time points, we obtained free and informed consent from our participants who were told that participation in the project was voluntary and that they could withdraw from the project at any time without any prejudice. A list of psychological help resources was provided to all participants at each time point to mitigate potential risks associated with participation in this project.

### Measures

#### Interpersonal emotional competence

To assess emotional competence at T1, we relied on the interpersonal subscales of the French version of the Profile of Emotional Competence (PEC; Brasseur et al., 2013). These subscales measure participants’ interpersonal emotional competence on five dimensions, namely identification (‘I often take the wrong attitude to people because I am not aware of their emotional state’), expression (‘I feel uncomfortable if people tell me about their problems, so I try to avoid it’), comprehension (‘I can easily explain the emotional responses of the people around me’), regulation (‘If someone came to me in tears, I would not know what to do’), and utilization (‘I know what to do to motivate people’). Each subscale includes five items answered on a 5-point Likert-type scale ranging from 1 (not at all) to 5 (completely). In this study, negatively worded items were reverse coded to obtain a positive score of emotional competence. We relied on a global factor of interpersonal emotional competence encompassing the five dimensions measured by the PEC, which exhibited adequate composite reliability as measured by McDonald’s (1970) omega coefficient or ω (ω = .91; see the online supplements for more details on the estimation of this global factor).

#### Help-seeking intentions

To measure social support–seeking intentions at T1, we adapted the General Help-Seeking Questionnaire (GHSQ; Wilson et al., 2005). This questionnaire was originally developed to assess social support–seeking intentions towards different social agents (intimate partner, friend, parent, other relative/family member, mental health professional, phone helpline, doctor/GP, teacher, minister/religious leader, and other) in the event of a personal or emotional problem. In this study, we relied on a modified 10-item version of the GHSQ. This version included help-seeking intentions towards members of the close social circle, namely intimate partner, friend, parent, and other relative/family member, to which we added ‘students in my program’ as an additional item for this category. This modified version also included help-seeking intentions towards professionals, namely mental health professional, phone helpline, and doctor/GP. We added ‘teacher’ and ‘other staff member at school’ to this list of professionals. Moreover, as there was no French version of the GHSQ when we initiated this study, our team used the back translation method to translate the modified 10-item version of the GHSQ from English to French. A professor and a graduate student in psychology whose first language is French generated the translated items. An undergraduate psychology student and a graduate education student translated the items back into English, and our team ensured that the original meaning had remained unchanged. Using this French version, participants were asked to reflect on their likelihood of asking members from each category of social agents (social circle and professionals) for help in the upcoming weeks in the event of a personal or school-related problem, using a 7-point Likert-type scale ranging from 1 (extremely unlikely) to 7 (extremely likely). Both factors (help-seeking from social circle and help-seeking from professionals) exhibited adequate composite reliability (ω = .68 and ω = .85, respectively; see the online supplements for more details on the estimation of these factors).

#### Educational success

To measure educational success at T2, we used the original French version of the School Success Questionnaire in VT (SSQ-VT; Tsakpinoglou & Véronneau, 2022). This 24-item scale assesses three distinctive dimensions of success according to the global mission of Quebec’s education system. These dimensions are instruction (4 items), qualification (10 items), and socialization (10 items). To respond to each item, participants relied on a 6-point Likert-type scale ranging from 1 (not at all) to 6 (completely). Sample items include ‘I have the feeling of having forgotten several skills that I have learned during my VT program’ (instruction), ‘The training I received helps me do my job well’ (qualification), and ‘I take into consideration the point of view of others’ (socialization). In this study, we relied on a modified version of the SSQ-VT which included four additional items for the instruction dimension (e.g. ‘I remember important information I learned during my training’) with the goal of improving its reliability which was found to be lower than for other dimensions in the validation study (Tsakpinoglou & Véronneau, 2022). Thus, in total, this dimension included eight items. Negatively worded items were reverse coded to obtain a positive score of educational success. We relied on three factors of educational success, one for each dimension measured by the SSQ-VT, all of which exhibited adequate composite reliability (Instruction: ω = .92; Qualification: ω = .96; Socialization: ω = .93; see the online supplements for more details on the estimation of these factors).

Additionally, we relied on a success-to-attempts ratio to provide an external assessment of our participants’ educational success at T2 based on school transcripts provided by the VT centres. Because transcripts in VT do not include grades (e.g. letters or percentages) but rather pass or fail mentions for each course, Dubeau et al. (2017) developed this ratio to obtain a continuous measure of academic achievement from these transcripts. This success-to-attempts ratio was calculated by dividing the number of courses passed by the number of courses attempted over the duration of each participant’s VT training. A ratio of 1 reflects the absence of failure while a ratio below 1 indicates that the participant failed and had to repeat one or more courses.

### Analytical approach

#### Preliminary analyses

Preliminary factor analyses were conducted to evaluate the structure and psychometric properties of all measures (except for the success-to-attempts ratio). Factor scores estimated in standardized units (*M* = 0, *SD* = 1) were saved from these preliminary measurement models and used as inputs in the main analyses (for a discussion on the advantages of factor scores over manifest scores, see Morin et al., 2016). Details on these models are reported in the online supplements. We estimated the bivariate correlations between all our variables with SPSS 29.

#### Main analyses

We used structural equation modelling (SEM) to test the sequential associations between these variables (see Figure 1). These analyses were conducted with the maximum likelihood robust (MLR) estimator implemented in Mplus 8.8 (Muthén & Muthén, 1998). We used full information maximum likelihood (FIML; Enders, 2010) to handle missing data related to attrition (38.81%), which allowed us to include all participants in the analysis (*N* = 219). Supporting this decision, results from a MANOVA revealed no significant differences between participants who completed both time points versus those who only participated at T1 on all T1 variables (main effect; F [3, 213] = 1.838, *p* = .141; Wilk’s Λ = .975). To assess the goodness-of fit of our model, we used the recommended goodness-of-fit indices (Hu & Bentler, 1999; Marsh et al., 2005): the comparative fit index (CFI), the Tucker–Lewis index (TLI), the root mean square error of approximation (RMSEA), and the standardized root mean square residual (SRMR). Adequate and excellent model fit are, respectively, indicated by CFI and TLI >.90 and .95, and by RMSEA and SRMR <.08 and .06.

## Results

**Figure 2.**
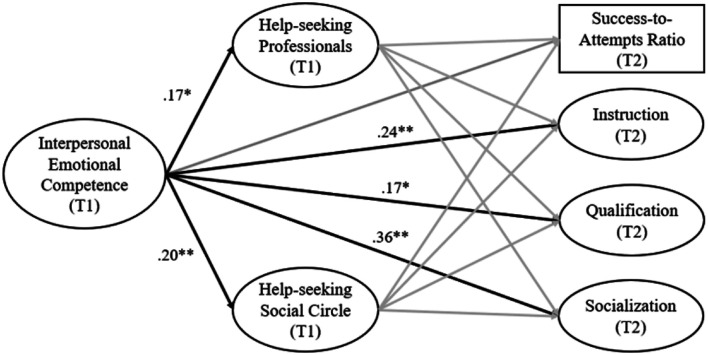
Significant results from the prediction model tested in this study. *Note.* Grey paths are not statistically significant. Sex was added in this model as a control variable in the prediction of educational success, but itis not illustrated in the figure for simplicity. Ovals: factor scores; rectangle: manifest score; T1: Time 1; T2: Time 2. *p < .05; **p < .01.

The bivariate correlations between all variables included in the study are presented in Table 1. The results from SEM are reported in Table 2 and illustrated in Figure 2. The tested model demonstrated adequate fit to the data: *X*^*2*^(2) = 2.042, *p* = .360; RMSEA = .010; SRMR = .017; CFI = 1.00; TLI = .998. In support of our first hypothesis, interpersonal emotional competence was a significant predictor of all three indicators of self-reported educational success measured at T2 (*βs =* .17 to .36, *ps* = .00–.04) but not of the success-to-attempts ratio. It was also a significant predictor of both help-seeking intentions towards the social circle (*β =* .20, *p* < .01) and towards professionals (*β =* .17, *p* = .02) as measured at T1. However, help-seeking intentions were not significantly related to any indicator of educational success at T2, which did not support our second hypothesis and prevented us from testing our third hypothesis (mediation analysis). These results were obtained while controlling for participants’ sex, which was significantly related to qualification (*β =* .25, *p* < .01) and to the success-to-attempts ratio (*β =* .15, *p* = .04), with male students exhibiting higher scores on these two variables.

**Table 1.** Correlations between the variables included in the study.

	1.	2.	3.	4.	5.	6.	7.	8.
1. Sex (0 = female, 1 = male)	**-**							
2. Emotional competence (T1)	−.060	**-**						
3. Help-seeking: social circle (T1)	−.061	.204**	**-**					
4. Help-seeking: professionals (T1)	−.102	.166*	.411**	**-**				
5. Success-to-attempts ratio (T2)	.133	−.003	.067	.136	**-**			
6. Instruction (T2)	.141	.237**	−.013	.049	.375**	**-**		
7. Qualification (T2)	.238**	.182*	.095	.026	.331**	.716**	**-**	
8. Socialization (T2)	.020	.385**	.102	.150	.145	.710**	.590**	-
Mean	-	3.35	4.26	2.62	.98	5.00	4.90	5.00
*SD*	-	.53	1.35	1.31	.07	.72	.94	.71

*Note*. Factor scores estimated in standardized units (*M* = 0, *SD* = 1) were used in all our analyses, apart from the success-to-attempts ratio. T1: Time 1; T2: Time 2. * *p* < .05; ** *p* < .01.

**Table 2.** Results from the predictive model.

Predictive paths	*B*	*SE*	*P*	*β*
IEC (T1) to Help-Seeking Intentions (T1)				
IEC → Social circle	.057	.020	.004	**.204**
IEC → Professionals	.155	.067	.021	**.167**
IEC and Sex (T1) to Educational Success (T2)				
IEC → Success-to-Attempts Ratio	−.002	.005	.650	−.031
IEC → Qualification	.218	.106	.039	**.171**
IEC → Instruction	.360	.109	.001	**.237**
IEC → Socialization	.269	.053	.000	**.362**
Sex → Success-to-Attempts Ratio	.023	.011	.038	**.146**
Sex → Qualification	.641	.217	.003	**.246**
Sex → Instruction	.441	.260	.090	.142
Sex → Socialization	.053	.121	.657	.035
Help-Seeking Intentions (T1) to Educational Success (T2)				
Social Circle → Success-to-Attempts Ratio	.013	.023	.567	.046
Social Circle → Qualification	.439	.435	.313	.096
Social Circle → Instruction	−.331	.455	.466	−.061
Social Circle → Socialization	.040	.226	.861	.015
Professionals → Success-to-Attempts Ratio	.010	.009	.270	.117
Professionals → Qualification	−.038	.149	.799	−.028
Professionals → Instruction	.051	.161	.752	.031
Professionals → Socialization	.056	.075	.457	.070

*Note*. Statistically significant coefficients are in bold. Sex was coded 0 (female) and 1 (male). IEC: Interpersonal Emotional Competence; T1: Time 1; T2: Time 2.

## Discussion

The current study sought to evaluate the role of interpersonal emotional competence and help-seeking intentions in predicting VT students’ educational success. We postulated that students’ interpersonal emotional competence, as measured during the first half of their training (T1), would be directly and positively linked to their self-reported indicators of educational success (instruction, qualification, socialization) and to their success-to-attempts ratio as measured at the end of their training (T2) (Hypothesis 1). We also postulated that students’ emotional competence would be indirectly linked to these indicators of educational success by fostering their help-seeking intentions towards members of their social circle and relevant professionals when encountering a personal or school-related problem during their training (Hypotheses 2 and 3). Overall, only our first hypothesis was fully supported as interpersonal emotional competence was the only variable significantly related to students’ educational success.

### Interpersonal emotional competence

Our results showed that VT students exhibiting greater interpersonal emotional competence reported higher levels of educational success in terms of instruction, qualification, and socialization. This suggests that students who can effectively identify, understand, manage, and express emotions in their relationships and interactions with others were more likely to believe that their training helped them develop the necessary skills to become knowledgeable, technically qualified, and socially competent workers. More precisely, it appears that interpersonal emotional competence facilitated the acquisition of knowledge and practical skills during our participants’ VT (the instruction component) as well as the ability to directly transfer and apply their knowledge and skills in the workplace (the qualification component). In line with our results, previous studies (e.g. Aloia & Solomon, 2015; Friedman et al., 2003) showed that interpersonal emotional competence facilitated the acquisition of skills such as teamwork, conflict resolution, effective communication, and open-mindedness, all related to the socialization component of educational success, which is crucial to developing and maintaining positive relationships in the workplace. Overall, these results are aligned with those obtained among various student populations (e.g. Arsenio & Loria, 2014; Denham et al., 2003, 2015; Shields et al., 2001) and show that the benefits of emotional competence for educational success are generalizable to VT students.

It is noteworthy however that the positive associations of interpersonal emotional competence with self-reported educational success were not replicated with the success-to-attempts ratio outcome. This might be explained by our decision of focusing on the interpersonal dimension of emotional competence, whereas a global measure (including both interpersonal and intrapersonal skills) may be a better predictor of external indicators of educational success. In fact, to obtain good grades and succeed in a specific course, students must be able to regulate their own emotions, especially when facing stressful situations (e.g. examination period) but also to implement and maintain effective learning strategies (Austin et al., 2010; Webster & Hadwin, 2015). Intrapersonal emotional competence might be particularly important when considering that VT programs are intensive and thus require a lot of self-discipline and organization from students. However, another and potentially more plausible explanation for this lack of significant association is the fact that the success-to-attempts ratio exhibited low variability, with most participants presenting a ratio of 1 (reflecting the absence of failures). Still, it is important to keep in mind that Quebec’s VT centres provide pass or fail mentions instead of grades. Therefore, the success-to-attempts ratio used in this study represented the most effective way of externally assessing the academic performance of our participants. Although such objective measures of achievement (e.g. grades) are usually recommended in the literature to differentiate students in terms of academic performance (York et al., 2015), they appear to be less relevant for VT students.

Interestingly, despite this low level of variability, the success-to-attempts ratio was positively correlated with the instruction and qualification components of educational success, meaning that the more students felt knowledgeable and qualified at the end of their training, the more they were likely to have succeeded in all their courses (and vice versa). The success-to-attempts ratio thus helps validate students’ self-perceptions of their academic abilities. Still, the moderate magnitude of these correlations (instruction: *r* = .38; qualification: *r* = .33) suggests that these variables represent different constructs that are not interchangeable. Given the dichotomous nature (pass or fail) of grades in VT, and their focus on the ‘instruction’ dimension, our results show that measuring several components of educational success (instruction, qualification, socialization) leads to a more nuanced and richer portrait of VT students’ educational success. Such portrait is likely to be relevant for understanding the factors that facilitate the integration of these students into the labour market (Voyer & Véronneau, 2022).

Another important result obtained in this study concerns the fact that interpersonal emotional competence predicted students’ help-seeking intentions, both towards members of their social circle and also towards professionals. This is aligned with previous research showing that the interpersonal component of emotional competence promotes greater social support–seeking (e.g. Ciarrochi & Deane, 2001; Keefer, 2015; Lei & Pellitteri, 2017). Numerous factors can explain these associations. First, individuals high in emotional competence are generally more comfortable discussing their problems with others, and they also tend to have more satisfying relationships, both of which increase their willingness to ask for help if needed (Rickwood et al., 2005). Moreover, being able to manage others’ emotions, which is a core component of interpersonal emotional competence, has been shown to positively predict help-seeking intentions through better past help-seeking experiences (Ciarrochi & Deane, 2001). In other words, the more interpersonal emotional skills individuals have, the more likely they are to have had positive and useful help-seeking experiences in the past, thereby increasing their willingness to seek help again in the future.

However, even though students with greater interpersonal emotional competence reported being more willing to seek informal and formal help during their training if needed, this willingness did not translate into higher levels of educational success by the end of their training. This result is surprising given that research conducted among various student populations has shown that social support is positively linked to academic performance (Demaray et al., 2005; Malecki & Demaray, 2003; Wu et al., 2023). We can only speculate on why help-seeking intentions were not related to educational success in the case of VT students.

First, although help-seeking intentions are assumed to be a major determinant of help-seeking behaviours (according to the theory of planned behaviour, Bosnjak et al., 2020; Nagai, 2015), past studies have shown that the magnitude of the association between these two constructs is generally modest and varies according to help sources (it is usually stronger when the help sources are members of the close social circle; Rickwood et al., 2005). It is thus possible that students who did encounter a personal or school-related problem during their training did not, in fact, ask for help, which limited the predictive power of help-seeking intentions on educational success. Many factors can explain the mismatch between help-seeking intentions and help-seeking behaviours, especially with regard to professionals. These factors include fear of stigmatization, lack of resources (e.g. money, time, transportation, and familiarity with the help-seeking process), lack of professional availability (e.g. mental health professionals and school staff), negative attitudes towards health professionals, and isolation (Aguirre Velasco et al., 2020; Rickwood et al., 2005). A phenomenon of help-negation has also been reported in previous studies conducted among various student populations, with individuals exhibiting high levels of psychological distress being less likely to seek formal and informal help for their difficulties (Ennis et al., 2019; Turner et al., 2023; Yakunina et al., 2010).

Moreover, among the students who did encounter a personal or school-related problem during their training and sought help for that specific problem, it is possible that their help-seeking behaviours were not oriented towards the appropriate source of support, thus cancelling their effects on educational success. For example, some students might have asked their friends or family members for help rather than their teachers for a school-related problem, thereby preventing them from receiving the necessary support. This would be aligned with past observations showing that most VT students do not identify school staff members as potential sources of support whereas most of them report that they can count on their family and friends when facing a problem (Beaucher et al., 2021). Together, our results raise important questions as to how the help-seeking process unfolds among VT students. More research is needed to investigate and document the help-seeking habits of this population as well as their implications for students’ educational success. Particular attention should be paid to barriers that prevent students’ intentions to seek help from translating into actual behaviours.

### Practical implications

In terms of practical implications, results from this study highlighted the role played by VT students’ interpersonal emotional competence in fostering their educational success, thus positioning these skills as key targets for interventions at various levels. From the student perspective, although emotional competence develops through a complex interaction between genetics, socialization, and cognitive development (Saarni et al., 2006), it also requires practice, and having opportunities to learn and apply emotional skills can greatly contribute to one’s emotional development (Guerra et al., 2014). Several intervention programs (in person or online) aimed at improving emotional competence have been developed and demonstrated their effectiveness among adult populations (for a meta-analysis and systematic review, see Hodzic et al., 2018; Kotsou et al., 2019), including university students (Gilar-Corbí et al., 2018). The components of these interventions could be integrated in VT schools in numerous ways. For instance, they could be used by the professionals assigned to supporting the well-being of students in VT schools (e.g. social workers and guidance counsellors) to help enhance the socio-emotional development of students who receive their services.

Beyond specific intervention programs, researchers have proposed that socio-emotional skills should be taught and practiced throughout the curriculum, just like other work skills related to students’ future jobs, to ensure that students are fully equipped to succeed in their studies and in their transition into the workplace (Ciarrochi & Mayer, 2013; Jennings & Greenberg, 2009; Schonert-Reichl, 2017). From a teaching and learning perspective, the interventions described above could therefore be used to enrich the curriculum offered by VT schools by integrating some of their main components into specific courses. Moreover, some pedagogical practices are known to facilitate students’ socio-emotional development. These practices include establishing a positive and predictable classroom environment, promoting positive teacher–student relationships, and using collaborative learning to allow students to learn the skills needed to get along with others (Dusenbury et al., 2015). VT teachers could be encouraged to rely on such practices for which professional development programs already exist (e.g. Guay et al., 2020).

Lastly, from a political and institutional perspective, our results reinforce the importance of considering VT students’ socio-emotional learning when developing or refining educational legislation and learning standards. Socio-emotional learning has gained in popularity over the last three decades, with frameworks such as the Collaborative for Academic, Social, and Emotional Learning (CASEL; Newman & Dusenbury, 2015) being developed to inform administrators, school leaders, and policymakers about the optimal conditions for fostering students’ socio-emotional development. These conditions involve creating specific policies and organizational structures that encourage and facilitate the implementation of evidence-based interventions aiming at cultivating various socio-emotional skills such as those described in this study (Dusenbury et al., 2015).

### Limitations and future directions

This study is founded on notable strengths, including the population under study, as VT students’ educational success has received little attention compared to other student populations. In addition, the prospective design and the inclusion of an external assessment of students’ educational success are methodological strengths that contribute to the validity of our results. In particular, we found that in the absence of grades in the students’ report cards, using the success-to-attempts ratio as a depending variable is not ideal because of the lack of variability. Yet, the significant correlation between this external measure and the educational success questionnaire provides convincing evidence that the latter is an adequate assessment of students’ success in spite of the known limitations of self-reported measures. Yet, our findings should be interpreted in light of certain limitations that must be addressed in future research.

First, the size of our sample was small, which may have masked some interesting results by limiting statistical power. Second, the success-to-attempts ratio that we used to assess our participants’ educational success with an external measure (as a complement to self-reports) exhibited a low level of variability, which could have precluded the obtention of significant results in relation to emotional competence and help-seeking intentions. As our results highlighted the importance of the instruction, qualification, and socialization components of VT students’ educational success, future research is needed to evaluate the role of these components in the prediction of important outcomes related to students’ integration into the workforce (e.g. occupational success, work engagement, and satisfaction). A third limitation is that our preliminary analyses (see the online supplement) led us to rely on a global factor of interpersonal emotional competence and on two factors of help-seeking intentions (social circle and professionals) in our main analyses. Although this allowed for a parsimonious prediction model, which was important given the size of our sample, it may have hidden some specificities that could be important for intervention. It would be interesting to replicate the present findings with a larger sample while also relying on a more fine-grained assessment of emotional competence (i.e. measuring emotional skills separately) and help-seeking intentions (i.e. measuring help sources separately).

Fourth, as our study only included help-seeking intentions, it was not possible to assess the relationships between these intentions and help-seeking behaviours. Future research is needed to verify whether students with the highest help-seeking intentions are indeed those who eventually need support, or if such intentions might rather be highest in well-functioning students who end up succeeding in their academic program without ever facing any major difficulty that would require external help. A better understanding of whether and how VT students’ help-seeking intentions translate into help-seeking behaviours is needed. It would also be useful to identify the factors that can facilitate or hinder this process among this specific population (e.g. barriers to help-seeking, help sources, and types of problems). Such research is particularly important considering that our results failed to support our hypothesis that help-seeking intentions would mediate the association between emotional competence and educational success. Lastly, given the important disparities regarding the structure and implementation of VT programs around the world, future research is needed to replicate the current findings among VT students from other countries and cultural backgrounds. This is even more important considering the paucity of research on the academic implications of emotional competence in VT students.

## Conclusion

Our findings highlighted the benefits of interpersonal emotional competence for the educational success of VT students, thus reinforcing the importance of supporting these students in the development and strengthening of their emotional skills. Although students’ emotional competence also predicted their help-seeking intentions towards members of their social circle and from professionals, these intentions were not associated with educational success. Future research is needed to both replicate the present findings and take further steps towards more deeply understanding the help-seeking process among VT students.

## Supplemental Material

Supplemental Material - Emotional competence and help-seeking intentions as predictors of educational success in vocational training students Supplemental Material for Emotional competence and help-seeking intentions as predictors of educational success in vocational training students by William Gilbert, Dale M Stack, Annie Dubeau, Lisa A Serbin, and Marie-Hélène Véronneau in Journal of Adult and Continuing Education

## Data Availability

The datasets generated during and/or analyzed during the current study are available on request from the corresponding author.*

## Note

1. For a review of how VT systems are organized and implemented across the world, and their relative importance in the overall educational system across countries, see OECD, 2015a.
