# Supplementary material for: Emotional competence and help-seeking intentions as predictors of educational success in vocational training students
Source: J Adult Contin Educ. 2024 Jul 24;30(2):617–39. doi: 10.1177/14779714241265463 (PMC13086227; doi:10.1177/14779714241265463)
Supplement: Supplemental Material - Emotional competence and help-seeking intentions as predictors of educational success in vocational training students [file sj-pdf-1-adu-10.1177_14779714241265463.pdf]

Online Supplements for:

Emotional competence and help-seeking intentions as predictors of educational success in vocational training students

Authors' note:

These online technical appendices are to be posted on the journal website and hot-linked to the manuscript. We would also be happy to have some of these materials brought back into the main manuscript, or included as published appendices if you deem it useful. We developed these materials to provide additional technical information and to keep the main manuscript from becoming needlessly long.

Preliminary Measurement Models

To verify the factorial structure and psychometric properties of our measures, we estimated a series of preliminary measurement models using the weighted least squares mean- and variance-adjusted estimator (WLSMV) in Mplus 8.8 (Muthén & Muthén, 2017). This estimator has been found to be superior to maximum-likelihood estimation for ordered-categorical items, particularly when the response categories follow asymmetric thresholds (for a review, see Finney & DiStefano, 2013). First, we relied on the bifactor-exploratory structural equation modeling framework (bifactor-ESEM; Morin et al., 2016) to evaluate the structure of the interpersonal subscales of the Profile of Emotional Competence (PEC; Brasseur et al., 2013). This choice was based on a recent study showing that this multidimensional questionnaire is best represented via bifactor-ESEM compared to alternative analytical strategies such as confirmatory factor analysis (CFA) and bifactor-CFA (Pirsoul et al., 2021). The bifactor-ESEM framework allows the estimation of a global (G-) factor, defined by all items, along with specific (S-) factors reflecting the variance in each dimension of a measure left unexplained by the G-factor. While the G-factor encompasses the commonalities (i.e., the common core) present among the items of a measure, the ESEM component allows conceptually relevant cross-loadings between the S-factors. For multidimensional scales, this combination (i.e., bifactor-ESEM) has been shown to promote more accurate model parameters than traditional approaches such as CFA (Asparouhov et al., 2015; Morin et al., 2019). The bifactor-ESEM model of emotional competence that was tested in this study (see Figure 1) included one G-factor reflecting participants' overall levels of interpersonal emotional competence and five S-factors reflecting each emotional competency measured by the PEC (identification, expression, comprehension, regulation, and utilization). However, to make sure that this analytical strategy provided the best results for the PEC, we estimated and contrasted three alternative models: a CFA model and ESEM model with five factors, and a bifactor-CFA model with five S-factors and one G-factor.

Next, we relied on CFA to evaluate the structure of the School Success Questionnaire in vocational training (SSQ-VT; Author et al., 2022) and of the General Help-Seeking Questionnaire (GHSQ; Wilson et al., 2005). The estimated model for the SSQ-VT included three factors reflecting distinctive

components of educational success, namely instruction, qualification, and socialization. The estimated model for the GHSQ included a factor reflecting help-seeking intentions towards members of the close social circle (intimate partner, friend, parent, non-parent family, another student) and a second factor reflecting help-seeking intentions from professionals (mental health professional, phone help line, doctor, teacher, another school staff member). For each tested model, the goodness-of fit was evaluated using recommended goodness-of-fit indices (Hu & Bentler, 1999; Marsh et al., 2005): The comparative fit index (CFI), the Tucker-Lewis index (TLI), and the root mean square error of approximation (RMSEA). Adequate and excellent model fit are respectively indicated by CFI and TLI >.90 and .95, and by RMSEA < .08 and .06.

Results

The fit indices of each tested model are presented in Table S1 while the parameter estimates of these models are presented in Table S2. For the PEC, the bifactor-ESEM model exhibited the best fit indices and was thus retained for further interpretation and analyses. In this model, the G-factor of emotional competence was well-defined (λ = .178 to .673; M_{λ} = .452; ω = .913) and offered a good representation of each of the five emotional competencies measured by the PEC (identification: λ = .402 to .636, M_{λ} = .541; expression: λ = .306 to .480, M_{λ} = .404; comprehension: λ = .292 to .609, M_{λ} = .458; regulation: λ = .442 to .673, M_{λ} = .533; utilization: λ = .178 to .534, M_{λ} = .325). In contrast, most S-factors kept little to no specificity once the effects of the G-factor were taken into account (ω = .435 to .812; M_{ω} = .543), meaning that specific emotional skills were no longer meaningful once the global levels of interpersonal emotional competence were considered. Because of that, and since the G-factor provides a much more parsimonious operationalization of emotional competence than the joint consideration of all specific facets covered by the PEC, we only retained the G-factor for our main analyses. For the SSQ-VT and GHSQ, the CFA model also demonstrated adequate fit indices. Results from these models revealed well-defined factors of educational success (instruction: λ = .252 to .894, M_{λ} = .685, ω = .922; qualification: λ = .442 to .941, M_{λ} = .751, ω = .958; socialization: λ = .546 to .907, M_{λ} = .682, ω = .923) and of help-seeking intentions (social circle: λ = .301 to .689; M_{λ} = .544; ω = .684; professionals: λ = .701

to .779; M_λ = .725; ω = .847). Factor scores (standardized units; *M* = 0, *SD* = 1) were saved from these models and used as inputs in the main analyses (for a discussion on the advantages of factor scores over manifest scores, see Morin et al., 2016).

References used in this supplement

Asparouhov, T., Muthén, B., & Morin, A.J.S. (2015). Bayesian Structural equation modeling with cross-loadings and residual covariances: Comments on Stromeyer et al. *Journal of Management*, *41*, 1561-1577.

Brasseur, S., Grégoire, J., Bourdu, R., & Mikolajczak, M. (2013). The profile of emotional competence (PEC): Development and validation of a self-reported measure that fits dimensions of emotional competence theory. *PloS one, 8*(5), e62635.

Finney, S.J., & DiStefano, C. (2013). Non-normal and categorical data in structural equation modeling. In G.R. Hancock, & R.O. Mueller (Eds.). *Structural equation modeling: A second course* (pp. 439-492). (2nd ed.). Information Age.

Hu, L.T., & Bentler, P.M. (1999). Cutoff criteria for fit indexes in covariance structure analysis: Conventional criteria versus new alternatives. *Structural Equation Modeling*, *6*, 1-55.

Marsh, H.W., Hau, K.-T., & Grayson, D. (2005). Goodness of fit evaluation in structural equation modeling. In A. Maydeu-Olivares & J. McArdle (Eds.), *Contemporary Psychometrics* (pp. 275-340). Erlbaum.

Morin, A. J., Arens, A. K., & Marsh, H. W. (2016). A bifactor exploratory structural equation modeling framework for the identification of distinct sources of construct-relevant psychometric multidimensionality. *Structural Equation Modeling: A Multidisciplinary Journal, 23*, 116–139.

Morin, A.J.S., Myers, N.D., & Lee, S. (2019). Modern factor analytic techniques: Bifactor models, exploratory structural equation modeling (ESEM) and bifactor-ESEM. In G. Tenenbaum & R. C. Eklund (Eds.), *Handbook of sport psychology* (4th ed.). Wiley.

Muthén, L. K. and Muthén, B. O. (1998–2017). *Mplus User's Guide* (8th ed.). Muthén & Muthén.

Pirsoul, T., Parmentier, M., & Nils, F. (2021). One step beyond emotional intelligence measurement in the career development of adult learners: A bifactor exploratory structural equation modeling framework. *Current Psychology*, 1-17.

Wilson, C. J., Deane, F. P., Ciarrochi, J. V., & Rickwood, D. (2005). Measuring help seeking intentions: properties of the general help seeking questionnaire. *Canadian Journal of Counselling*, *39*(1), 15-28.

Figure S1

Graphical Representation of the Bifactor-ESEM Model of Emotional Competence

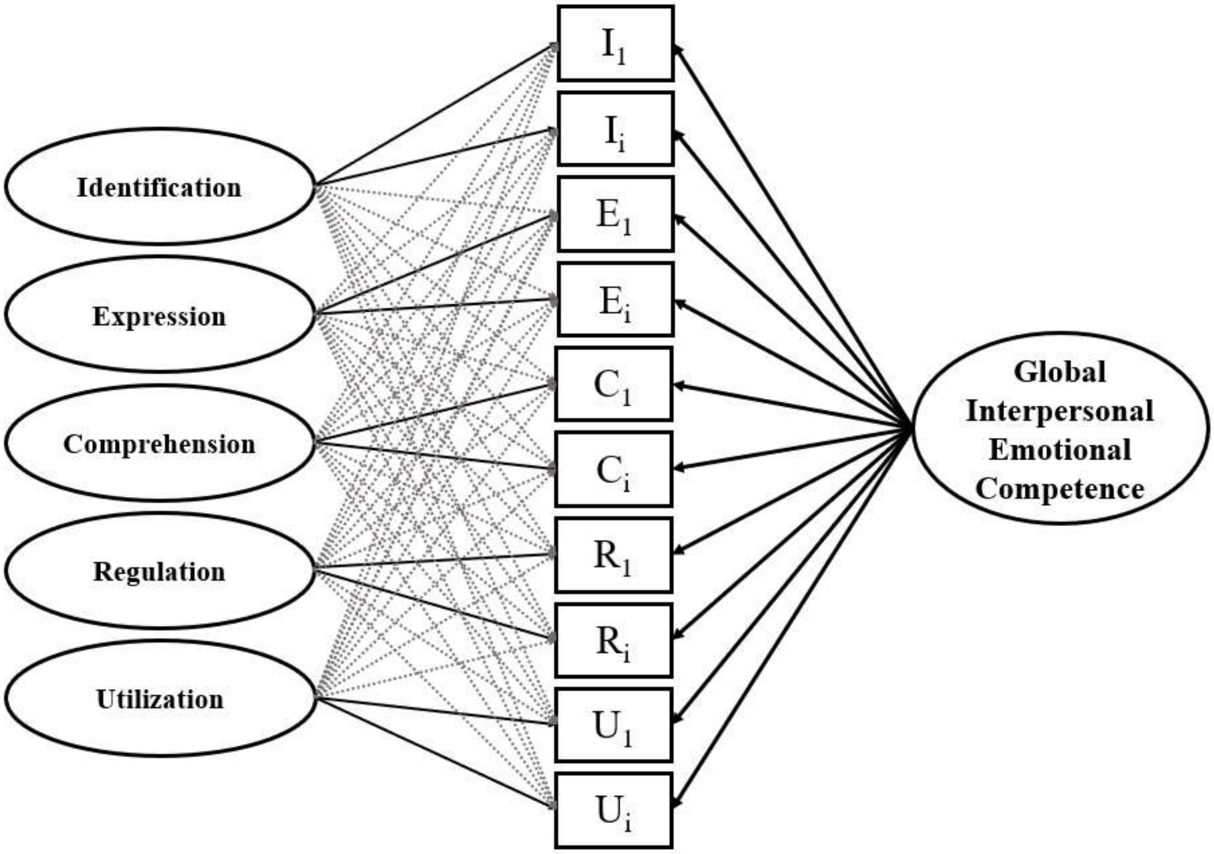

Note. Ovals represent latent factors while rectangles represent items. Full arrows represent target loadings while dashed arrows represent cross-loadings. I: Identification; E: Expression; C: Comprehension; R: Regulation; U: Utilization; i: items 2 to 5 of each subscale.

Table S1

Goodness-of-Fit Statistics for the Estimated Measurement Models

Description	χ^2	*df*	CFI	TLI	RMSEA
Profile of emotional competence (interpersonal)					
CFA	925.713*	265	.723	.686	.107
ESEM	266.867*	185	.966	.944	.045
Bifactor-CFA	776.012*	250	.779	.735	.098
Bifactor-ESEM	217.608*	165	.978	.960	.038
School success questionnaire in vocational training					
CFA	616.354*	347	.927	.920	.076
General help-seeking questionnaire					
CFA	72.233*	33	.955	.939	.074

Note. χ^2 = Chi-square test of exact fit; df = Degrees of freedom; CFI = Comparative fit index; TLI = Tucker-Lewis index; RMSEA = Root mean square error of approximation; SRMR = Standardized Root Mean Square Residual. * $p < .05$.

Table 2

Standardized Parameter Estimates from the Retained Measurement Models

PEC	Global EC (λ)	I (λ)	E (λ)	C (λ)	R (λ)	U (λ)	δ
Identification (I)							
I1	**.461****	**-.347****	.077	.259**	-.053	-.034	.590
I2	**.636****	**.264****	.182*	-.073	-.043	-.306**	.392
I3	**.635****	**-.360****	-.080	.122	-.025	-.071	.439
I4	**.402****	**.252***	-.055	-.095	-.284**	-.039	.681
I5	**.570****	**.185**	-.203**	.037	-.332**	-.109	.476
ω		.435					
Expression (E)							
E1	**.484****	-.114	**.435****	.231**	.171**	.046	.552
E2	**.306****	.244**	**.191***	-.216*	-.081	-.356**	.368
E3	**.323****	-.020	**.440****	-.371**	-.265**	-.300*	.456
E4	**.480****	-.064	**.597****	.084	.233**	.030	.705
E5	**.428****	.207*	**.238****	-.076	-.136	-.130*	.545
ω			.588				
Comprehension (C)							
C1	**.346****	.433**	-.288**	**.063**	.099	-.211*	.479
C2	**.609****	-.140	-.042	**.476****	-.007	-.115*	.630
C3	**.507****	-.117	-.076	**.516****	-.029	.011	.405
C4	**.292****	.272*	-.161*	**-.233**	-.027	-.234**	.346
C5	**.535****	.040	.185*	**-.171**	-.251**	-.199**	.677
ω				.448			
Regulation (R)							
R1	**.442****	.077	.049	.141*	**.379****	-.003	.633
R2	**.549****	-.067	.108	-.201**	**-.049**	-.142*	.619
R3	**.673****	-.107	.128**	-.027	**.576****	-.024	.186
R4	**.496****	-.307**	-.129*	-.094	**.248***	.294**	.486
R5	**.505****	-.068	-.036	.073	**.171***	.025	.704
ω					.435		
Utilization (U)							
U1	**.178***	-.090	.043	-.019	-.045	**.838****	.254
U2	**.387****	.193**	-.126*	.061	.170*	**.658****	.332
U3	**.339****	.050	-.214**	.125*	.116	**.683****	.341
U4	**.534****	-.220	-.123	-.051	.475**	**.038**	.422

U5	**.185***	-.191*	-.231**	.007	-.038	**.609****	.504
ω	.913					.812	
SSQ-VT	I (λ)	S (λ)	Q (λ)				δ
Instruction (I)							
I1	**.850****						.277
I2	**.894****						.201
I3	**.448****						.800
I4	**.668****						.553
I5	**.794****						.370
I6	**.853****						.272
I7	**.723****						.477
I8	**.252****						.937
ω	.923						
Qualification (Q)							
Q1		**.817****					.332
Q2		**.793****					.372
Q3		**.856****					.267
Q4		**.712****					.493
Q5		**.615****					.622
Q6		**.442****					.805
Q7		**.760****					.422
Q8		**.930****					.134
Q9		**.644****					.585
Q10		**.941****					.115
ω		.958					
Socialization (S)							
S1			**.610****				.628
S2			**.716****				.487
S3			**.584****				.659
S4			**.907****				.178
S5			**.710****				.497
S6			**.699****				.511
S7			**.733****				.463
S8			**.555****				.692
S9			**.546****				.702
S10			**.764****				.417

ω	.926

GHSQ	SC (λ)	P (λ)	δ
Social circle (SC)			
SC1	**.310****		.904
SC2	**.579****		.664
SC3	**.689****		.526
SC4	**.662****		.562
SC5	**.481****		.768
ω	.684		
Professionals (P)			
P1		**.720****	.481
P2		**.717****	.486
P3		**.779****	.393
P4		**.701****	.508
P5		**.706****	.502
ω		.847	

Note. EC: Emotional competence; SS: School success; λ: Factor loading; δ: Item uniqueness; ω: model-based omega composite reliability based on McDonald (1970); Target factor loadings are in bold. * $p < .05$; ** $p < .01$.
